# Safety of Surgery after Neoadjuvant Targeted Therapies in Non-Small Cell Lung Cancer: A Narrative Review

**DOI:** 10.3390/ijms222212244

**Published:** 2021-11-12

**Authors:** Tomasz Marjanski, Robert Dziedzic, Anna Kowalczyk, Witold Rzyman

**Affiliations:** 1 Thoracic Surgery Department, Medical University of Gdansk, Sklodowskiej-Curie 3A, 80-211 Gdansk, Poland; dziedzic@gumed.edu.pl (R.D.); wrzyman@gumed.edu.pl (W.R.); 2 Department of Oncology and Radiotherapy, Medical University of Gdansk, Sklodowskiej-Curie 3A, 80-211 Gdansk, Poland; anna.kowalczyk@gumed.edu.pl

**Keywords:** lung cancer, non-small cell lung cancer, surgery, neoadjuvant therapy, EGFR-TKIs, ALK-TKIs, safety, complications, toxicity, adverse events

## Abstract

New drugs, including immune checkpoint inhibitors and targeted therapy, have changed the prognosis in a subset of patients with advanced lung cancer, and are now actively investigated in a number of trials with neoadjuvant and adjuvant regimens. However, no phase III randomized studies were published yet. The current narrative review proves that targeted therapies are safe in neoadjuvant approach. Unsurprisingly, administration of therapy is related to an acceptable toxicity profile. Severe adverse events’ rate that rarely compromises outcomes of patients with advanced lung cancer is not that commonly accepted in early lung cancer as it may lead to missing the chance of curative surgery. Among those complications, the most important factors that may limit the use of targeted therapies are severe respiratory adverse events precluding the resection occurring after treatment with some anaplastic lymphoma kinase and rarely after epidermal growth factor receptor tyrosine kinase inhibitors. At this point, in the presented literature assessing the feasibility of neoadjuvant therapies with anaplastic lymphoma kinase and epidermal growth factor receptor tyrosine kinase inhibitors, we did not find any unexpected intraoperative events that would be of special interest to a thoracic surgeon. Moreover, the postoperative course was associated with typical rate of complications.

## 1. Introduction

Targeted therapies and immunotherapy are the cornerstones of progress in the treatment of advanced lung cancer [1,2,3]. In the era of lung cancer screening, there is a potential for increased incidence of early disease [4,5]. Adverse events of novel neoadjuvant protocols as well as their implications on surgical outcomes are crucial. Surgery is a gold standard in the treatment of non-small-cell lung cancer (NSCLC) being twice as effective in terms of overall survival (OS) when comparing to stereotactic body radiation therapy [6]. Nevertheless, all kinds of ablative therapies should still undergo assessment within clinical trials.

Surgical resection leads to 80–90% of 5-year OS in the IA1-IA3 stages of NSCLC. Poorer results in patients treated in stages IB (73%), IIA (65%), IIB (56%), IIIA (41%), and IIIB (24%) stimulate further efforts for the implementation of complex treatment protocols [7]. In a LACE meta-analysis of individual data of 4584 patients, an adjuvant chemotherapy led to improvement of OS by 5.4% (hazard ratio (HR) 0.89, 95% confidence interval (CI) 0.82–0.96) and disease-free survival (DFS) by 5.8% (HR 0.84 95%, CI 0.78–0.91) [8]. A systematic review of 32 randomized trials involving more than 10,000 participants compared the timing of chemotherapy (neoadjuvant versus adjuvant) and did not demonstrated an advantage of either strategy (OS HR 0.99, 95% CI 0.81–1.21; DFS HR 0.96, 95% CI 0.77–1.20) [9]. Another systematic review of 15 randomized controlled trials including 2385 patients who underwent neoadjuvant chemotherapy disclosed a benefit in terms of improved survival by 5% (HR 0.87, 95% CI 0.78–0.96) [10]. Although equivalent efficacy of preoperative and postoperative chemotherapy strategies are suggested, postoperative chemotherapy remains the standard treatment. Authors suggest that perioperative chemotherapy regimens reached a plateau of their efficacy [8,9,10].

New drugs, including immune checkpoint inhibitors and targeted therapy, have changed the prognosis in a subset of patients with advanced NSCLC [1,2,3], and are now actively investigated in a number of trials with neoadjuvant and adjuvant regimens [11,12,13]. Immunotherapy with or without chemotherapy significantly improves survival of patients with advanced NSCLC depending on the programmed cell death ligand-1 (PD-L1) expression [14]. However, molecular subtypes of NSCLC with driver mutations may derive less benefit from immune checkpoint inhibitors [15,16]. Due to progress in molecular diagnostic technologies, a number of actionable genetic abnormalities can be found in NSCLC and a number of targeted agents are being used in advanced stages of the disease [17]. In NSCLC patients, epidermal growth factor receptor (EGFR) and anaplastic lymphoma kinase (ALK) alterations are most frequently found, especially in no or light smokers.

Activating mutations of epidermal growth factor receptor (EGFR)—exon 19 deletion and exon 21 substitution are present in 10–36% of Caucasian patients with adenocarcinoma of the lung [18,19,20]. Chromosomal rearrangements of ALK are diagnosed less commonly in 4–5% of patients and are not exclusive for adenocarcinoma [21,22]. The frequency of driver mutations in Asian patients is higher [23,24], while the data in other ethnicities are conflicting [25]. Diagnosis of these two groups of common molecular aberrations allows effective use of targeted therapies.

There are attempts to include targeted therapies into complex strategies of treatment of early NSCLC [12]. Toxicity profiles in the treatment of patients with advanced NSCLC are well documented [26,27]. Implementing novel agents not previously used preoperatively leads to new patient safety questions [28]. Safety issues reported in trials with neoadjuvant immunotherapy are monitored and are a matter of interdisciplinary discussion [29,30]. While offering targeted therapies in neoadjuvant settings, their influence on the perioperative period should raise the interest of thoracic surgeons. The key issue of the neoadjuvant approach is safety. Benefits of previously used neoadjuvant chemotherapy are limited [8]. If an unacceptably high complication rate or perioperative mortality is recorded during the trial, it reduces the benefit in OS, which may explain the absence of neoadjuvant therapies in the guidelines [31,32]. The new neoadjuvant therapies have a potential for increased rate of postoperative complications and escalated intraoperative complexity. Before involving the patient in a multidisciplinary treatment approach, other issues must be addressed, e.g., what would be the effect of the neoadjuvant treatment on the delay of the resection? How many patients would not qualify for resection due to progression and/or complications? Additional issues: thoracoscopy-open conversion rate, the extent of surgery, resectability rate, and quality of life will be answered as the body of evidence accumulates.

**Figure 1:**
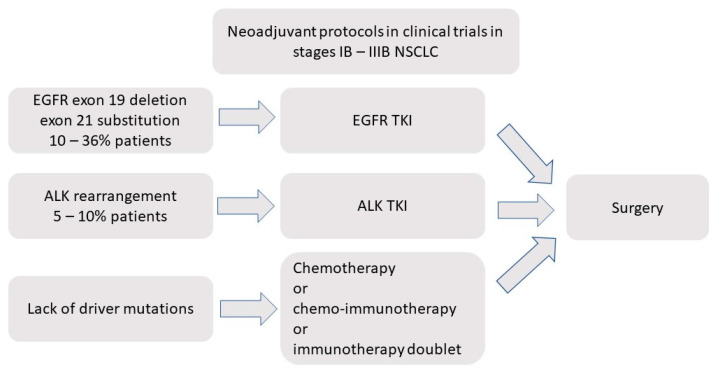
General study protocols of neoadjuvant approach in clinical trials. ALK-TKI anaplastic lymphoma kinase tyrosine kinase inhibitors, EGFR-TKI epidermal growth factor receptor tyrosine kinase inhibitors, NSCLC non-small cell lung cancer.

In clinical trials, the indications for EGFR and ALK tyrosine kinase inhibitors (TKIs) inhibitors are being extended beyond stage IV (Figure 1). Improved DFS in most of the trials with adjuvant EGFR-TKIs in EGFR mutant patients in stage IB-IIIA adenocarcinoma (for example ADAURA trial) promises the change of current practice in these patients [12,33,34,35,36]. Equal effect of neoadjuvant versus adjuvant chemotherapy on OS [8,9,10] is not observed in recently published studies with neoadjuvant and adjuvant targeted therapies [12,33,34,35,36,37,38,39,40,41,42,43,44,45]. However, different treatment protocols and foremost different EGFR mutation status of the patients treated in these trials precludes an exact comparison of the results. Patient’s adherence to the treatment, low neoadjuvant treatment discontinuation rate, low rate of grade 3/4 toxicities leading to significant delay of surgery, and low rate of progression on the neoadjuvant therapy are essential to eventually include targeted therapies in future studies assessing efficacy. Results of ongoing trials with third-generation EGFR-TKI (NeoADAURA NCT04351555, LAURA NCT03521154, NCT03433469) are awaited [46].

In this narrative review, we have analyzed and presented all available scientific papers on the topic of safety of surgery after neoadjuvant targeted therapies. The papers were extracted after searching electronic databases and scanning reference lists from relevant studies. The literature search was not restricted by the time of publication.

## 2. Typical Complications of EGFR-TKIs

The most common adverse events of administration of EGFR-TKIs in advanced NSCLC are skin toxicity, gastrointestinal toxicity, pulmonary toxicity, hepatic toxicity, and ocular toxicity [26,47,48,49]. EGFR-TKIs are generally well tolerated. However, administration of the drugs may be related to a few toxicities leading to treatment discontinuation and/or dose reduction due to poor patients’ adherence [47,48]. Grade 3/4 toxicities according to the Common Toxicity Criteria for Adverse Events (CTCAE) are reported to occur in 29% of patients treated with gefitinib, 54% of patients treated with erlotinib, and 42% of patients treated with afatinib. Toxicities lead to discontinuation of treatment in <10% of patients and are lower when comparing to chemotherapy [49,50]. Pulmonary toxicity is important from the point of view of eventual lung surgery because development of interstitial lung disease (ILD) being a consequence of administration of EGFR-TKIs significantly impairs physiological fitness [51]. Inadequate pulmonary reserve may result in resignation from surgery [52,53].

### 2.1. Skin Toxicity

High expression of EGFR in the basal layer of the skin leads to common skin toxicity due to EGFR-TKIs. The incidence of this complication is reported in 66–100% of patients [54,55]. Grade 3 skin toxicity is reported in 2–20% of patients [56]. The most common presentation are: rash, xerosis, erythema, fissures, telangiectasia, pruritus [56]. Most of the recommendations concerning the prevention and treatment of skin toxicity of EGFR-TKIs are based on clinical routine [26]. Topical and systemic use of corticosteroids and antibiotics, topical use of retinoids, vitamin K and emollients are the treatment options [26,54]. The adequate supportive treatment usually allows for relief of symptoms and continuation of treatment.

### 2.2. Gastrointestinal Toxicity

Impairment of the function of the basal layer of the epithelium of the tongue, esophagus, and intestines during treatment with EGFR-TKIs leads to a second common group of complications [57]. The most significant gastrointestinal adverse event, diarrhea is reported in 21–95% of patients [58]. The onset of diarrhea is usually present in the first few weeks of therapy. Diarrhea is more severe in patients treated with afatinib [59]. Usually loperamide and adequate hydration are needed. In some patients, dose reduction is indicated [60]. Other complications related to the mucosa of the gastrointestinal tract are stomatitis and mucositis [57]. The adverse events are usually manageable with adequate supportive care and eventually dose reduction of the drugs [26,58].

### 2.3. Pulmonary Toxicity

Pulmonary toxicity is a very rare complication of treatment with EGFR-TKIs; however, it may have important implications from the perspective of physicians planning multidisciplinary treatment. The most common presentations are interstitial lung disease (ILD), pneumonitis, pneumonia, and hemoptysis [61,62,63]. The incidence of any grade pulmonary toxicity is low and occurs in 0.7–4.0% of patients treated with various generation EGFR-TKIs for advanced NSCLC [62,64]. However, if this complication occurs, the mortality in EGFR-TKI-induced ILD or pneumonitis is as high as 36% [65]. The mechanism of pulmonary injury is debated and there are is no causative treatment [26,66]. Risk factors for the development of ILD are preexisting ILD, male sex, older age, history of tobacco smoking, worse performance status [67]. The symptoms characteristic for rapid onset of ILD comprise of worsening dry cough and dyspnea within days to weeks [68,69]. The diagnosis is made on the basis of chest X-ray or high-resolution computerized tomography of the chest after exclusion of pneumonia and cancer progression [70]. Negative cultures obtained in bronchoalveolar lavage obviously do not preclude infectious origin of pulmonary infiltrates, which may require molecular testing for various pathogens responsible for interstitial pneumonia. Treatment discontinuation and steroids are the only interventions offered when ILD after EGFR-TKIs occurs [26,69].

### 2.4. Cardiac Complications

Even 10% of patients treated with osimertinib and 5% of patients treated with first- and second-generations EGFR-TKIs may be diagnosed with QT interval prolongation [71]. QT prolongation is usually labeled as grade 1 or 2 toxicity and usually does not lead to life-threatening cardiac arrhythmias [64].

### 2.5. Treatment-Related Mortality

Lethal toxicity of targeted therapies is rare. In advanced-stage NSCLC, the administration of gefitinib, erlotinib, and afatinib is related to deaths in 2.3%, 0.8%, and 1.1%, respectively. The most common cause of death is pneumonitis (65%) [48,49].

## 3. Neoadjuvant EGFR-TKIs in Early NSCLC

There is a limited experience with neoadjuvant administration of EGFR-TKIs in early NSCLC. No phase III randomized studies were published. Most of the studies deliver a low level of evidence from single-arm trials [37,38,43,44] and retrospective observations [41,42]. We refer only to two small randomized controlled trials directly comparing the neoadjuvant therapies with the use of erlotinib vs. gemcitabine/cisplatin chemotherapy [39,40]. The study protocols evolved as the body of evidence for the efficacy of EGFR-TKIs grew. Primarily, the patients were enrolled in the trials regardless of the presence of driver mutations [40,44]. Further, only patients with tumors harboring sensitizing ex19del/L858R mutations were included in observational or interventional studies [37,39,40,42].

The rate of pathological complete response (pCR) after neoadjuvant use of EGFR-TKIs is low (0–12%) [37,38,39]. Major pathological response (MPR) is noted in 8–24% of patients [38,43,47]. The results of MPR and pCR rates are worse in the case of EGFR-TKIs than with immune checkpoint inhibitors reported recently [72]. Relatively low pathological response rates after neoadjuvant EGFR-TKIs may be a result of wild-type tumors in some studies and a high rate of not radical resections (5–50%) [40,42]. These shortcomings may be corrected by meticulous preoperative qualification of patients and elaborating the study protocol. Neoadjuvant treatment should not aim to downstage tumor characterized by marginal resectability. The primary aim should be improving the OS and DFS by reducing distant relapse instead of reducing the number of local recurrences. On the other hand, limited duration of neoadjuvant therapy may result in insufficient exposition to the EGFR-TKIs. Whether this factor is responsible for better results of adjuvant [12] versus neoadjuvant trials will be answered by future research.

The safety of the EGFR-TKIs in the preoperative setting is high. The toxicity profile of neoadjuvant EGFR-TKIs is the same as in the case of therapy in an advanced stage. Most of the studies report the number of perioperative complications (Table 1). No intraoperative events were mentioned in the analyzed studies. Postoperative mortality was 0%. The complications after surgery were typical and their level was low. Deterioration of pulmonary function after neoadjuvant administration of erlotinib was reported in 2 studies where it was observed in 3–5% of patients [39,44]. In one retrospective study with the use of different agents, 7% of the resections were complicated by chylothorax, which is higher than commonly reported in surgical databases [73,74]. This high rate of complications may be a result of a small study group.

## 4. Typical Complications in ALK Inhibitors

Treatment with ALK TKIs is usually well tolerated and grade 3/4 toxicities are rarely observed [76]. The most commonly reported adverse events affect the digestive tract, lungs, and heart [77]. Fatigue, visual disorders, peripheral oedema, and neurological disturbances are also common [76,77]. Observed toxicities lead to discontinuation of treatment in 5–12% of patients with NSCLC [78,79]. There are differences in the toxicity profiles between different ALK inhibitors. Gastrointestinal and hepatic toxicities are common during treatment with ceritinib, visual disorders with crizotinib, edema, myalgias and dysgeusia with alectinib, and respiratory complications probably with brigatinib [77].

### 4.1. Gastrointestinal and Hepatic Complications

Toxicities related to the alimentary tract are very common but usually mild. Diarrhea, nausea, vomiting, and constipation are reported in up to 55% of patients. Grade ≥3 toxicities are observed in less than 3% [79,80,81]. Nausea, vomiting, and diarrhea rates are highest with ceritinib and lowest with alectinib. Constipation occurs most commonly with crizotinib and alectinib comparing to brigatinib and ceritinib. Liver toxicity is usually limited to biochemical changes and acute liver injury with clinical implications is extremely rare [27]. All grades transaminase elevation is reported in 19–33% of patients treated with ALK-TKIs [79,80,81,82] with grade ≥3 occurring in less than 5% of patients [27,78]. Liver toxicity is more frequent in patients treated with ceritinib and crizotinib than with alectinib or brigatinib.

### 4.2. Pulmonary Complications

Pulmonary toxicity is of significant concern when neoadjuvant therapy is considered, especially in the case of lung cancer surgery. Upper respiratory infections are observed in patients receiving crizotinib and alectinib. Cough is noted in 25% patients receiving brigatinib and 16% of patients receiving crizotinib [80]. However, in the case of underlying advanced NSCLC, it may be difficult to differentiate drug-related adverse events from symptoms of the disease itself. ILD occurs in 0–3% of patients [27,80]. In the review of the four PROFILE trials with crizotinib, the incidence rate of drug-related ILD was assessed as 1.2% [83]. Typical pulmonary complications of brigatinib and ceritinib are pneumonia and respiratory failure with the serious adverse events (SAE) rate up to 14% [78]. Oppositely pulmonary adverse events are rarely observed in patients treated with other ALK-TKIs [84]. Interestingly, brigatinib-related ILD may occur rapidly, even within the first seven days of therapy [80,81].

### 4.3. Cardiac Complications

Cardiac arrhythmias are the most common complications of ALK-TKIs [85]. Bradycardia and QTc prolongation are transient and rarely lead to cardiac insufficiency [85]. Disfunction of the left ventricle is not reported [86]. Death as a result of arrhythmias has rarely been recorded [27].

### 4.4. Treatment-Related Mortality

Treatment-related mortality is uncommon and was reported in 0.9% of patients [27]. The most important reasons for treatment-related mortality are pulmonary complications, e.g., ILD, pneumonitis, and pneumonia [80].

## 5. Neoadjuvant Treatment with ALK Inhibitors in Early NSCLC

The PROFILE 1014 study demonstrated improved objective response rate and progression-free survival in ALK-positive non-squamous NSCLC patients treated with crizotinib comparing to chemotherapy in first-line therapy [87]. Since then, ALK-TKIs became a standard of care in ALK-positive advanced NSCLC. Expanding the indications beyond advanced NSCLC led to the introduction of this therapy to neoadjuvant, induction, and adjuvant protocols in NSCLC. Positive results of trials comparing the second-generation ALK-TKIs–alectinib and brigatinib [80,88] are expected to lead to introduction these agents in individualized treatment protocols.

Neoadjuvant therapy aims to improve the OS by reducing the rate of distant relapse. The patients accepted for neoadjuvant treatment should be upfront resectable, as the local control is not the aim of this approach. In contrast, the downstaging-induction treatment is an approach which may be accepted only in a highly selected population of patients with locally advanced NSCLC. A number of published case studies present a downstaging rather than a neoadjuvant approach due to locally advanced disease [89,90]. It should be underlined that only carefully planned trials, where only patients with objectively resectable tumors are accepted, will aim to prove survival benefit. Currently, high-quality evidence for the neoadjuvant use of ALK inhibitors is lacking. In the largest series of patients operated on after neoadjuvant crizotinib pCR occurred in 18% of patients, pathological response in 91%, pathological nodal downstaging in 27%, and R0 resection rate in 91%. These results together are rationales for an ongoing phase II trial (NCT03088930).

Currently available, heterogeneous data demonstrate that neoadjuvant therapy with ALK-inhibitors is safe (Table 2). A low rate of treatment-related SAE and low rate of postoperative complications were reported. There was no perioperative mortality.

## 6. Final Remarks

Trials with neoadjuvant administration of immunotherapy or chemo-immunotherapy report much higher rates of obtained pathological responses than trials with neoadjuvant EGFR and ALK-TKIs (21–45% MPR and 7–15% pCR) [94]. Immunotherapy and chemotherapy combinations may lead to even higher rates of pathological response: MPR 57–83% and pCR 33–63% [73,95]. It is hypothesized that the immune checkpoint inhibitors are more efficient in the neoadjuvant approach comparing to adjuvant due to intact immune landscape of the primary tumor and regional lymph nodes. The presence of tumor accompanied by T-cell infiltration may promote an adaptive antitumor immune response. By inducing immune response before surgery, effective immune memory, targeting primary tumor and potential micrometastatic disease, can be generated to spread durable protection against cancer recurrence and distant metastases development [96]. This effect is not utilized in the mechanisms of action, neither of molecularly driven therapies nor chemotherapy. Molecularly driven therapies are limited by timedelaying the surgery. EGFR and ALK mutated tumors are characterized by a lower tumor mutation burden, they present lower numbers of antigens that generate less prominent immunological responses. Those tumors are less susceptible to the immune checkpoint inhibitors, which require constant development of effective therapies also in neoadjuvant protocols [95].

The current narrative review proves that molecularly driven therapies are safe in a neoadjuvant approach. Unsurprisingly, administration of therapy is related to an acceptable toxicity profile. However, the SAEs’ rate that rarely compromises outcomes of patients with advanced NSCLC is not that commonly accepted in early NSCLC, as it may lead to missing the chance of curative surgery. Among those complications, the most important factors that may limit the use of targeted therapies are the grade ≥3 respiratory adverse events precluding the resection [37], occurring after treatment with some ALK inhibitors and rarely after EGFR-TKIs.

Additionally, novel agents involved in neoadjuvant protocols raise concerns of unpredicted intraoperative difficulties (for example perihilar fibrosis). At this point, in the presented literature assessing the feasibility of neoadjuvant therapies with EGFR and ALK inhibitors, we did not find any unexpected intraoperative events that would be of special interest to a thoracic surgeon. Moreover, the postoperative course was associated with the typical rate of complications.

## Figures and Tables

**Table 1:** Toxicities of neoadjuvant EGFR-TKIs described in the literature. SAE, serious adverse event; EGFR-TKI, epidermal growth factor receptor tyrosine kinase inhibitor; NR, not reported; PAL, persistent air leak; GI, gastrointestinal; ALT, Alanine aminotransferase; AST, Aspartate aminotransferase.

Study	EGFR-TKI in Study Group	Number of Patients in Study Group	Delay of Surgery	EGFR-TKI Complications	Intraoperative Complications in EGFR-TKI Group	R0 Resection Rate in EGFR-TKI Group	Postoperative Complications in EGFR-TKI Group
Schaake et al., 2012 [44]	Erlotinib	60	24 days	Rash 61% Diarrhea 35% Pneumonitis 5%	0%	7% of patients found out to be unresectable	Pneumonia 2% PAL 2% Blood transfusion 3%
Han et al., 2012 [75]	Erlotinib	7	56 days	NR	0%	20% of resected patients	NR
Zhong et al., 2015 [40]	Erlotinib	24	6 weeks of treatment	Rash 100% Diarrhea 42%	0%	50%	0%
Tan et al., 2019 [43]	Gefitinib	14	At least 4 weeks of treatment	AST/ALT elevation 8%	NR	NR	NR
W.Z. Zhong et al., 2019 [39]	Erlotinib	37	6 weeks of treatment	Rash 76% Diarrhea 68% Deterioration of pulmonary function precluding surgery 3%	0%	73%	Arrhytmia 6% Lung infection 6% Poor wound healing 6% PAL 3% Pneumothorax 3%
Xiong et al., 2019 [37]	Erlotinib	19	56 days	Rash 26% 13% were not operated due to SAE	NR	68% of resected patients	NR
Lv et al., 2020 [42]	Different agents	43	8 weeks of treatment	NR	0%	95%	Chylothorax 7% Atelectasis 5% Arrhytmia 2%
Y. Zhang et al., 2021 [38]	Gefitinib	35	61 days	Skin toxicity 69% GI symptoms 49%	0%	12% of patients found out to be stage IV at surgery	Chylothorax 12%
Bao et al., 2021 [41]	Different agents	42	NR	NR	NR	NR	NR

**Table 2:** Toxicities of neoadjuvant ALK inhibitors reported in the literature. ALK, anaplastic lymphoma kinase; NR, not reported.

Study	ALK Inhibitor in Study Group	Number of Patients	Delay of Surgery	ALK Inhibitor Complications	Intraoperative Complications	Postoperative Complications
Tian et al., 2020 [89]	Crizotinib	1	12 weeks	Grade 1 hepatic damageMild edema	None	None
Kilickap et al., 2019 [90]	Crizotinib	1	6 weeks	NR	NR	NR
Xie et al., 2021 [91]	Gemcitabine, cisplatine, crizotinib	1	2 months	None	NR	NR
C. Zhang et al., 2019 [92]	Crizotinib	11	Median 41 days	Grade 4 hepatitis 9%	None	Pneumonia 9% Dyspnoe 9%
Imanishi et al., 2018 [93]	Alectinib	1	3 months	None	None	None

## Abbreviations

ALK	anaplastic lymphoma kinase
ALT	Alanine aminotransferase
AST	Aspartate aminotransferase
CTCAE	Common Toxicity Criteria for Adverse Events
DFS	disease-free survival
EGFR	epidermal growth factor receptor
HR	hazard ratio
GI	gastrointestinal
ILD	interstitial lung disease
MPR	major pathological response
NR	not reported
NSCLC	non-smallcell lung cancer
OS	overall survival
PAL	persistent air leak
pCR	pathological complete response
PD-L1	programmed cell death ligand-1
SAE	serious adverse event
TKI	tyrosine kinase inhibitors
